# Drawing a line?—Visuo-constructive function as discriminator between healthy individuals, subjective cognitive decline, mild cognitive impairment and Alzheimer’s disease and predictor of disease progress compared to a multimodal approach

**DOI:** 10.1007/s40211-022-00455-8

**Published:** 2023-02-01

**Authors:** Amelie Tokaj, Johann Lehrner

**Affiliations:** Department of Neurology, Medical University of Vienna, Währinger Gürtel 18–20, 1097 Vienna, Austria

**Keywords:** Dementia, Visuoconstruction, VVT 3.0 Screening, Clock Drawing Test, Mini Mental State Examination, Demenz, Visuokonstruktion, VVT 3.0 Screening, Uhrentest, Mini-Mental-State-Test

## Abstract

**Purpose:**

One cognitive domain impaired in Alzheimer’s disease (AD) is visuo-construction. The Vienna Visuo-constructional Test 3.0 Screening (VVT 3.0 Screening) measures this cognitive domain. This study examines how it works in the differentiation of AD from healthy controls (HC) and the prodromal stages subjective cognitive decline (SCD) and mild cognitive impairment (MCI) and also how it performs in prediction of progress compared to the Mini Mental State Examination (MMSE) and the Sunderland Clock Drawing Test (CDT).

**Methods:**

Data from 622 patients (33 HC, 68 SCD, 301 MCI, 220 AD) who completed all three tests were obtained. Furthermore, 117 patients were examined in a follow-up. Data were analyzed in a retrospective analysis comparing the validity of tests in diagnosis and prediction using receiver operator characteristic (ROC) curves and multinominal logistic regression.

**Results:**

The VVT 3.0 Screening shows some ability to discriminate between AD and all other participants (sensitivity: 62.1%, specificity: 83.1%), while of the three examined tests none was able to predict membership to all experimental groups or to predict disease-progress adequately. As the VVT 3.0 Screening is short, easy to apply and largely language independent, it can be considered an alternative to the MMSE in certain situations.

**Conclusions:**

The VVT 3.0 Screening is useful to discriminate between AD and all other participants and can be an alternative to the MMSE in certain situations.

## Abstract (de)

**Ziel:**

Eine kognitive Domäne, die zur Diagnostik der Alzheimer-Erkrankung genutzt werden kann, ist die Visuokonstruktion. Die Messung kann mittels Vienna Visuo-constructional Test 3.0 Screening (VVT 3.0 Screening) erfolgen. In der vorliegenden Studie wird untersucht, ob der Test die Alzheimer-Erkrankung (AD) von ihren Prodromalstadien subjektiver kognitiver Abbau (SCD), leichte kognitive Beeinträchtigung (MCI) und gesunden Kontrollen (HC) besser unterscheiden kann als der Uhrentest nach Sunderland (CDT) und der Mini-Mental-Status-Test (MMSE). Die drei Tests werden außerdem hinsichtlich ihrer Nützlichkeit zur Vorhersage der Krankheitsprogression verglichen.

**Methoden:**

Insgesamt 622 ProbandInnen (33 HC, 68 SCD, 301 MCI, 220 AD) absolvierten alle drei Tests. Davon nahmen 117 an einer Folgeuntersuchung teil. In einer retrospektiven Datenanalyse wurden Receiver-operating-characteristics(ROC)-Kurven sowie eine multinomiale logistische Regression eingesetzt, um die Validität der drei Tests in Diagnostik und Vorhersage zu bestimmen.

**Ergebnis:**

Der VVT 3.0 Screening kann zwischen AD und Nicht-AD unterscheiden (Sensitivität: 62,1 %, Spezifität: 83,1 %). Keiner der drei Tests unterschied jedoch zuverlässig alle Experimentalgruppen oder sagte den Krankheitsverlauf adäquat vorher. Der VVT 3.0 Screening als kürzerer und weniger von Sprachverständnis abhängige Test zeigt ausreichend Validität hinsichtlich der Fragestellung, um als Alternative zu gelten.

**Fazit:**

Der VVT 3.0 Screening ist nützlich, um Nicht-Alzheimer-PatientInnen von Alzheimer-PatientInnen zu unterscheiden und stellt in bestimmten Fällen eine Alternative zum MMSE dar.

## Introduction

Live expectancy in Austria and other Western countries continues to increase and with it the prevalence of age-related diseases like dementia [1]. As dementia can lead to hospitalization, disability, and death, it is a great (psychological) burden for patients and their relatives, as well as for the health care system [1, 2]. The most common cause of dementia is Alzheimer’s disease [1], an early diagnosis of which is recommended in current guidelines, as there is evidence that early therapy has better long-term effects and can reduce disease burden and need of care [2]. Also, early diagnosis is the foundation for valid research on the disease.

Alzheimer’s disease dementia (AD) does not begin with an incisive event but proceeds slowly and subtly. A means to divide this continuum into categories is to allocate patients into the groups healthy controls (HC), the prodromal stages subjective cognitive decline (SCD) and mild cognitive impairment (MCI) and AD [3]. SCD is a preclinical, transitional phase in a patient’s life where cognitive impairment cannot be objectified but patients experience a subjective decline in cognitive capability [3, 4]. This phase was found to be heterogeneous and difficult to characterize by objective characteristics [4]. The next prodromal phase—MCI—may progress to any form of dementia but also remain stable or improve. It is defined as a mild form of cognitive impairment that can be objectified using neuropsychological testing while the ability to perform tasks and activities of daily living is preserved, which is no longer the case once a patient converts to AD [3, 5]. The diagnosis of AD is usually performed clinically using neuropsychological testing, which is easy to apply, non-invasive and can give a fast overview of a patient’s cognitive status and monitor disease progression over time [2].

The Mini Mental Status Examination (MMSE) [6] is one test recommended for broad assessment of cognitive impairment and to evaluate the overall severity of dementia [2]. It is one of the tests used most frequently for dementia screening, and provides a sensitivity of up to 81% and a specificity of up to 89% in detection of dementia [7]. However, the MMSE was found not to be able to identify the predemential stages SCD or MCI nor reliably predict conversion to AD [8]. The MMSE has a lower sensitivity in detection of MCI than other broad neuropsychological screening tests [7, 9] and guidelines do not recommend it for MCI detection [2].

The Clock Drawing Test (CDT) is recommended for detection of dementia in combination with other tests but not as a singular instrument, it could not be shown to be reliable in discrimination of AD and prodromal phases [2, 9, 10]. Alternative scoring systems have been proposed to increase validity of the test and were shown to have some ability in longitudinal prediction of the conversion of MCI to dementia [11]. Neither of the two tests can render reliable diagnosis of prodromal stages or predict conversion to AD.

One of the domains that can be impaired in AD according to the National Institute on Aging and Alzheimer’s Association (NIA-AA) and the DSM‑5 is visuo-constructional ability [5, 12]. It could also be shown to deteriorate early within the course of the disease and be a valid means to discriminate between patients suffering from dementia and healthy controls [13–16] and in some cases also between MCI and HC [14]. Also, visuospatial function could be shown to be a predictor of fast cognitive decline [17] and to deteriorate faster than global cognition in individuals developing AD [16, 18]. This makes visuospatial function a sensible domain to test for when trying to detect early stages of neurocognitive impairment.

The Vienna Visuo-constructional Test 3.0 (VVT 3.0) integrates different visuo-constructional tests for the use in diagnosis of AD and its precursory states. It comprises a clock-copying task similar to the one used in the Montreal Cognitive Assessment (MoCa) [19], two overlapping pentagons as known from the MMSE [6] and a three-dimensional cube (as seen in the MoCa as well).^1^ The VVT 3.0 Screening can be used in the original version or a shortened screening version. In previous studies, the VVT 3.0 Screening could be shown to detect visuo-constructional impairment and to detect patients with AD, although it was not able to distinguish the four diagnostic groups HC, SCD, MSI, and AD [15, 20]. Similar results could be obtained for the shortened Vienna Visuo-constructional Test 3.0 Screening (VVT 3.0 Screening) [13]. The test is of special interest regarding the inconsistent performance of the MMSE and the Sunderland CDT in allocation of patients to different diagnostic groups. The subject of the current analysis was to compare the VVT 3.0 Screening to the MMSE and the CDT (Sunderland) in detecting HC, SCD, MCI, and AD and predicting progress.

## Methods

### Study design

The current study is a retrospective analysis of data that was obtained at the Department of Clinical Neurology of the Medical University of Vienna and was originally collected for the Vienna Conversion to Dementia Study from 2008–2017. All participants included in this study sought medical attention at the Department of Neurology at the Medical University of Vienna themselves or were referred by their primary physician. All participants had an initial neuropsychological assessment; a subgroup of participants attended a follow-up.

### Participants

Neurocognitive data from 622 patients (285 men and 337 women) was examined. Patients were included if aged 50 years or older, living in Austria, and presenting to the clinic for assessment of possible cognitive disorder. Individuals showing evidence of neurological or psychiatric comorbidities that might compromise cognition were excluded, as were data sets with relevant gaps. Relevant sociodemographic data on participants is shown in Table 1.

**Table 1:** Sociodemographic characteristics of diagnostic subgroups

Variable	HC	SCD	MCI	AD	∑
	*n* = 33	*n* = 68	*n* = 301	*n* = 220	*N* *=* *622*
Age (years) *** *Mdn* (IQR)	59.8 ± 7.7 58 (54–65.5)	69.6 ± 9.5 70.5 (63.5–76)	69.6 ± 9.2 71 (63.5–76.5)	74.2 ± 8.1 76 (70–79)	70.7 ± 9.4 73 (65–77)
Education (years)*** *Mdn *(IQR)	14.9 ± 4.4 14 (10.5–19)	12.6 ± 3.9 12 (9–16)	12.7 ± 4.2 12 (9–16)	11.0 ± 3.4 10 (8–13)	12.2 ±4
					12 (8–16)
Sex female	22 (66.7%)	34 (50.0%)	156 (51.8%)	125 (56.8%)	337 (54.2%)
Male	11 (33.3%)	34 (50.0%)	145 (48.2%)	95 (43.2%)	285 (45.8%)

*HC* Healthy controls, *SCD* Subjective cognitive decline, *MCI* Mild cognitive impairment, *AD* Alzheimer’s dementia, *Mdn* Median, *IQR* Interquartile range, Age and years of education are reported as mean ± standard deviation and Mdn (IQR), means were compared using Kruskal–Wallis test for age and years of education

***p < 0.001

**Fig. 1:**
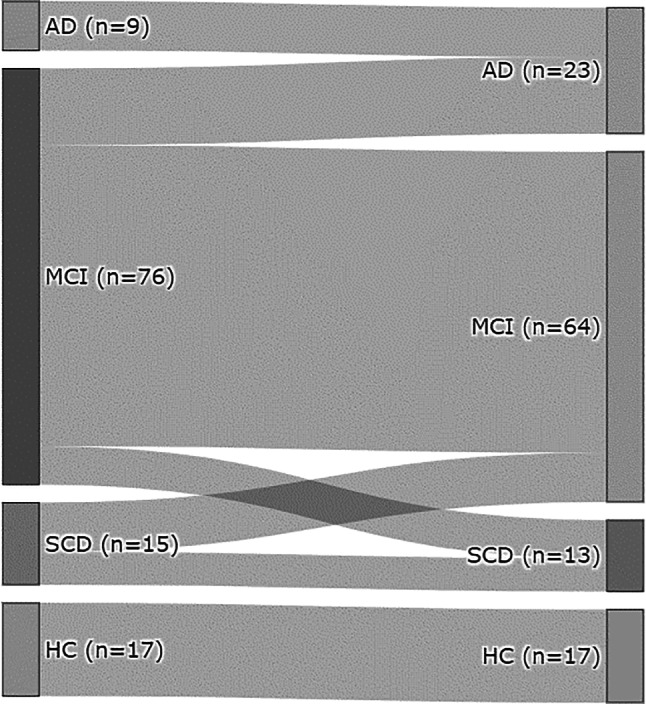
Sankey diagram of patient flow between measurements. Patient numbers at the first measurement are shown on the left (AD: *n* = 9, MCI: *n* = 76, SCD: *n* = 15, HC: *n* = 17); patient numbers at follow-up are shown on the right (AD: *n* = 23, MCI: *n* = 64, SCD: *n* = 13, HC: *n* = 17). Width of bar from left to right indicates number of patients changing from one group to another between measurements (HC to HC: *n* = 17, SCD to SCD: 6, SCD to MCI: 9, MCI to SCD: 7, MCI to MCI: 55, MCI to AD: 14, AD to AD: 9). *HC* Healthy controls, *SCD* Subjective cognitive decline, *MCI* Mild cognitive impairment, *AD* Alzheimer’s dementia

Based on their performance in the Neuropsychological Test Battery Vienna (NTBV) and an interview, patients were clinically diagnosed. The guidelines of Jessen et al. [21] were used for identification of SCD. For MCI and HC, the Mayo clinic criteria [22] were used; the NICDS-ADRDA [23] criteria were applied for AD. A total of 33 participants were diagnosed as healthy controls (HC), 68 persons as SCD, 301 participants as MCI, and 220 participants were diagnosed with AD. 117 patients agreed to participate in a follow-up examination (Fig. 1).

### Instruments

Participants completed a clinical interview, the Neuropsychological Test Battery Vienna (NTBV) [24], the Beck Depression Inventory (BDI-II) [25], the Wortschatz-Test (WST) [26], the MMSE [6], the CDT evaluated according to Sunderland [27], and the VVT 3.0 Screening. The latter consists of ten items—three for drawing a clock, three for drawing two pentagons, and four for drawing cubes. It takes about 2–3 min to administer and was shown to have comparable test quality as the long version [28]. It can be obtained from www.psimistri.com.

### Statistical analysis

Statistics were conducted using SPSS® 25 (IBM, Ehningen, Germany). Sankey diagram was drawn using Plotly graphic library for Python 3.9 within PyCharm Community edition 2021.2.3 (JetBrains, Munich, Germany). Level of significance was set at α = 5%; thus, results from hypothesis testing with *p* ≤ 0.05, according to type I error, are denoted significant. To interpret the practical relevance of results, effect sizes were calculated where appropriate.

As the sociodemographic and the neuropsychological test values (MMSE, VVT 3.0 Screening, and CDT) were skewed in both the cross-sectional and the longitudinal data set as indicated by Kolmogorov–Smirnov test in conjunction with values of standardized skewness, nonparametric tests were used and the alternative characteristics Mdn and IQR were calculated and are reported additionally to mean and standard deviation (SD)*.*

Kruskal–Wallis combined with pairwise comparisons with adjusted *p*-levels and Jonkheere’s test for trends were used to test for differences and trends between groups. For the dichotomous variable sex, the chi-squared (χ^2^) test was used.

The relationships between the three tests and between the variables age and education and each of the tests were explored using Spearman’s rank correlation. Bootstrapping was administered (*n* = 1000) and 95% confidence intervals (CIs) are reported.

To examine the three test’s validity in diagnosis of AD, receiver operating characteristics (ROC) were calculated with AD as positive condition. The area under the curve (AUD) was determined, as were sensitivity and specificity at optimal cut-off scores determined by the Youden index (YI). Positive and negative predictive values (PPV/NPV) were determined as were positive and negative likelihood ratios (LR+/LR−). Analogous ROC analyses were calculated for the discrimination of MCI and non-MCI excluding AD patients and SCD and non-SCD excluding the groups AD and MCI from analysis. Also, ROC curves were calculated within the longitudinal sample for the three test’s ability to discriminate between stable participants and progressors, setting progress as positive condition.

Subsequently, multinominal logistic regressions were performed to assess the VVT 3.0 Screening’s, the CDT’s and the MMSE’s ability to allocated participants to all four experimental groups. Pairwise comparisons using binary logistic regressions were used to compare single groups. To minimize the alpha (α) error for conduction of multiple comparisons, results were adjusted using the Bonferroni–Holm correction. Cohen’s kappa (κ) and pseudo‑R^2^ (Nagelkerke) were calculated as reliability measures.

Wilcoxon signed-rank test was used to assess the differences of neuropsychological test results between measurements. As progress in only one direction was assumed, one-tailed significance was calculated.

The nonparametric Mann–Whitney U test for independent samples was used to test for differences between the participants that returned for the second measurement and those dropping out as well as to test for differences between participants whose disease had converted into a different experimental group to those who remained stable. To assess progression between test appointments, McNemar–Bowker test was used.

## Results

### Differences between groups

**Fig. 2:**
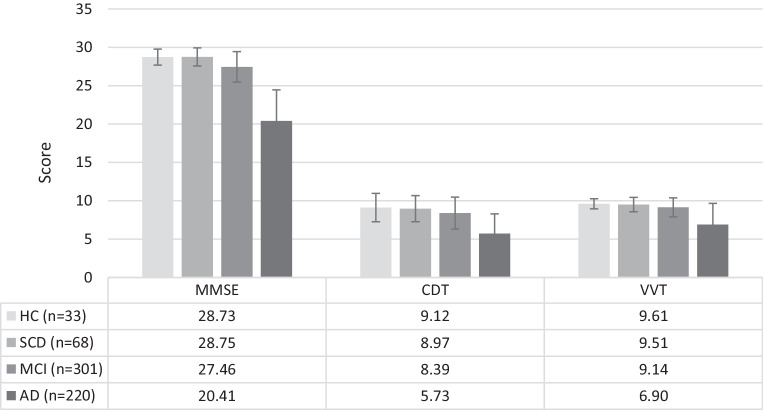
Bar chart of scores of participants by group in Mini Mental Status Examination (MMSE), Sunderland Clock Drawing Test (CDT), and Vienna Visuo-constructional Test 3.0 Screening (VVT 3.0 Screening) including error bars indicating standard error. MMSE scores are shown on the left (HC: 28.73, SCD: 28.75, MCI: 27.46, AD: 20.41), CDT scores are shown in the middle (HC: 9.12, SCD: 8.97, MCI: 8.39, AD: 5.73), VVT 3.0 Screening scores on the right (HC = 9.61, SCD: 9.51, MCI: 9.14, AD: 6.90). *HC* Healthy controls, *SCD* Subjective cognitive decline, *MCI* Mild cognitive impairment, *AD* Alzheimer’s dementia

Cross-sectional comparison of neuropsychological test results showed significant differences in performance between the four diagnostic groups HC, SCD, MCI, and AD (Table 2, Fig. 2). The diagnostic groups differed significantly in MMSE score (H (3) = 402.74, *p* < 0.001), in CDT score (H (3) = 173.55, *p* < 0.001), and in VVT 3.0 Screening score (H (3) = 167.37, *p* < 0.001). Jonckheere’s test revealed significant results for all three tests (*p* < 0.001 for all tests).

**Table 2:** Neuropsychological performance of diagnostic subgroups

	HC	SCD	MCI	AD	∑
Test procedure	*n* = 33	*n* = 68	*n* = 301	*n* = 220	*n* *=* *622*
MMSE (0–30)*** *Mdn* (IQR)	28.73 ± 1.04 29 (28–30)	28.75 ± 1.18 29 (28–30)	27.46 ± 1.99 28 (26–29)	20.41 ± 4.05 21 (19–23)	25.2 ± 4.53 27 (23–29)
CDT (0–10)*** *Mdn* (IQR)	9.12 ± 1.85 10 (9–10)	8.97 ± 1.70 10 (9–10)	8.39 ± 2.08 9 (8–10)	5.73 ± 2.56 5 (4–8)	7.55 ± 2.6 9 (5–10)
VVT 3.0 Screening (0–10)*** *Mdn* (IQR)	9.61 ± 0.66 10 (9–10)	9.51 ± 0.94 10 (9–10)	9.14 ± 1.24 10 (9–10)	6.90 ± 2.76 8 (5–9)	8.41 ± 2.2 9 (8–10)

*HC* Healthy controls, *SCD* Subjective cognitive decline, *MCI* Mild cognitive impairment, *AD* Alzheimer’s dementia, *MMSE* Mini Mental Status Examination, *CDT* Clock drawing test, *VVT 3.0 Screening* Vienna Visuo-constructive Test 3.0 Screening, *Mdn* median, *IQR* interquartile range, scores are reported as mean ± standard deviation and Mdn (IQR), Kruskal–Wallis test was performed to assess differences in data

***p < 0.001

### Ability of tests to differentiate groups

**Fig. 3:**
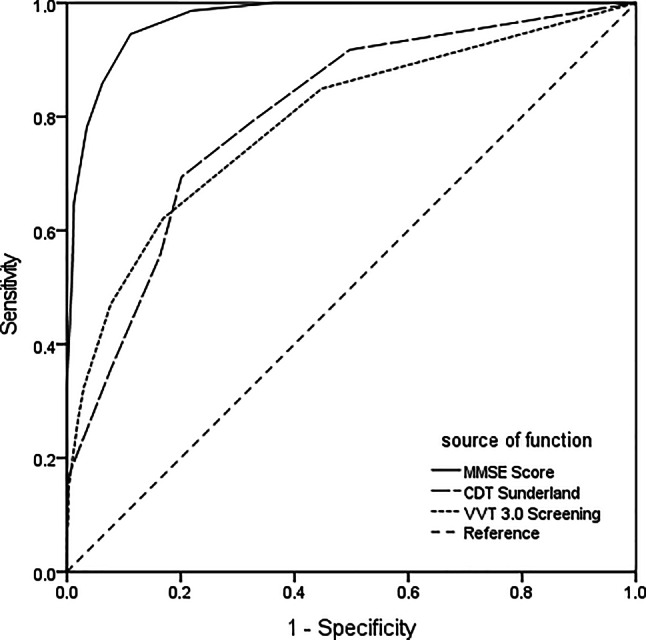
Receiver operator characteristic (ROC) curves for Mini Mental Status Examination (MMSE), Sunderland Clock Drawing Test (CDT) and the Vienna Visuo-constructional Test 3.0 Screening (VVT 3.0 Screening) with Alzheimer’s dementia as positive condition. A higher area under the curve (AUC) indicating high diagnostic validity. AUC for MMSE was 0.972, for VVT 3.0 Screening 0.791, and for CDT 0.804

To determine the ability of tests to differentiate between the AD group and all other participants, ROC curves with AD as positive condition including AUC were calculated: Using this method, sensitivity and specificity of a test method are set in relation and a cut-off score balancing both values can be determined. The AUC can have values from 0–1, a value of 0.5 meaning test performance is equal to random allocation of participants. For the MMSE, the AUC for discrimination of AD and non-AD was 0.972 and an optimal cut-off score of 25.5 points with a sensitivity of 94.5% and a specificity of 88.8% was calculated (YI = 0.833, LR+ = 8.444, LR− = 0.0617, PPV = 0.880, NPV = 0.929). Nagelkerke’s R^2^ as a measure of adequate prediction of patient group was calculated as 0.80; Cohen’s κ was 0.80 (*p* < 0.001), which means that 80% of variance of diagnosis could be explained by the predictor. The AUC measured for the CDT pursuing the same question was 0.804 at optimal cut-off of 7.5 points (sensitivity 69.4%, specificity 79.9%, YI = 0.493, LR+ = 3.445, LR− = 0.383, PPV = 0.65, NPV = 0.78). For the CDT, Nagelkerke’s R^2^ was 0.33, while Cohen’s κ was 0.41 (*p* < 0.001). AUC for the VVT 3.0 Screening was 0.791 at optimal cut-off score of 8.5 points, providing a sensitivity of 62.1% and a specificity of 83.1% (YI = 0.452, LR+ = 3.671, LR− = 0.456, PPV = 0.77, NPV = 0.76). For the VVT 3.0 Screening Nagelkerke’s R^2^ was 0.34; Cohens κ was 0.43 (*p* < 0.001). With a shift of the cut-off to 9.5, the sensitivity of the VVT 3.0 Screening reached 84.9%, while specificity was 55.2% (see Fig. 3).

Subsequently, ROC curves were plotted excluding AD setting MCI as positive condition (*n* = 402) to see if the tests could differentiate between MCI patients and non-MCI patients (SCD and HC). For the MMSE, the AUC was 0.700 and an optimal cut-off of 28.5 points (sensitivity 65.4%, specificity 68.3%, YI = 0.338). Based on this, LR+ was 2.066, LR− was 0.506, while PPV was 0.86 and NPV was 0.39 (Nagelkerke’s R^2^ = 0.15, Cohen’s κ = 0.273, *p* < 0.001). For the CDT, an AUC of 0.599 was found at an optimal cut-off of 9.5 points (sensitivity 54.2%, specificity 63.4%, YI = 0.175, LR+ = 1.478, LR− = 0.723, PPV = 0.815, NPV = 0.317, Nagelkerke’s R^2^ = 0.29, Cohen’s κ = 0.132, *p* = 0.002). For the VVT 3.0 Screening, an AUC of 0.6 and an optimal cut-off of 9.5 points was determined, which lead to a sensitivity of 49.2% and a specificity of 68.3% (YI = 0.175. LR+ = 1.55, LR− = 0.744, PPV = 82.2, NPV = 31.1, Nagelkerke’s R^2^ = 0.41, Cohen’s κ = 0.125, *p* = 0.002).

The ROC curves excluding MCI patients and setting SCD as positive condition (*n* = 101) to determine whether SCD could be differentiated from HC rendered an AUC of 0.476 for MMSE, an AUC of 0.561 for CDT, and an AUC of 0.509 for VVT 3.0 Screening. No further analyses were conducted.

The additional multinomial logistic regression models were significant (*p* < 0.001) for all three tests (MMSE: χ^2^ = 594.49, Cohen’s κ = 0.55, Nagelkerke’s R^2^ = 0.689; CDT: χ^2^ = 181.49, Cohen’s κ = 0.30, Nagelkerke’s R^2^ = 0.284; VVT 3.0 Screening: χ^2^ = 191.67, Cohen’s κ = 0.28, Nagelkerke’s R^2^ = 0.297) for the entire sample with HC as reference category. The results of the pairwise group comparisons for classification into each diagnostic group and for each test are specified in Table 3. All regression models only used the two categories MCI and AD. For the MMSE, group membership was correctly predicted for 74.9% of the whole patient sample (92% correct MCI, 85.9% correct AD). The CDT predicted the correct diagnostic group for 60.7% of patients (80.1% of MCI patients, 62.1% AD patients). Using the VVT 3.0 Screening, 60.6% of patients were correctly assigned, 90.7% of MCI patients and 47.3% of AD patients.

**Table 3:** Parameters of multinomial logistic regression analysis

	Reference category		B (SE) intercept	B (SE) MMSE	Odds ratio [95% CI]	*p*
MMSE	HC (33)	SCD (68)	0.308 (4.842)	0.014 (0.169)	1.015 [0.729; 1.412]	0.932
		MCI (301)	16.790 (4.156)	−0.517 (0.145)***	0.596 [0.449; 0.792]	< 0.001***
		AD (220)	40.672 (4.701)	−1.495 (0.170)***	0.224 [0.161; 0.313]	< 0.001***
	SCD (68)	MCI (301)	15.970 (3.064)	−0.514 (0.107)***	0.598 [0.486; 0.738]	< 0.001***
		AD (220)	56.621 (11.622)	−2.132 (0.439)***	0.119 [0.050; 0.280]	< 0.001***
	MCI (301)	AD (220)	23.851 (2.209)	−0.977 (0.089)***	0.376 [0.316; 0.448]	< 0.001***
CDT	HC (33)	SCD (68)	1.297 (1.293)	−0.063 (0.141)	0.939 [0.713; 1.236]	0.652
		MCI (301)	4.268 (1.127)	−0.234 (0.122)	0.791 [0.623; 1.006]	0.056
		AD (219)	7.027 (1.126)	−0.664 (0.124)***	0.515 [0.404; 0.657]	< 0.001***
	SCD (68)	MCI (301)	2.928 (0.713)	−0.166 (0.079)	0.847 [0.726; 0.989]	0.035***
		AD (219)	6.138 (808)	−0.651 (0.093)***	0.522 [0.435; 0.626]	< 0.001***
	MCI (301)	AD (219)	2.825 (0.319)	−0.439 (0.042)***	0.645 [0.594; 0.700]	< 0.001***
VVT	HC (33)	SCD (68)	2.351 (2.859)	−0.170 (0.298)	0.843 [0.471; 1.511]	0.567
		MCI (301)	7.706 (2.506)	−0.584 (0.261)***	0.558 [0.335; 0.930]	0.025***
		AD (220)	12.424 (2.521)	−1.187 (0.264)***	0.305 [0.182; 0.511]	< 0.001***
	SCD (68)	MCI (301)	4.939 (1.515)	−0.369 (0.159)***	0.691 [0.506; 0.945]	0.020***
		AD (220)	10.758 (1.751)	−1.094 (0.187)***	0.335 [0.232; 0.483]	< 0.001***
	MCI (301)	AD (220)	4.799 (0.599)	−0.613 (0.069)***	0.542 [0.474; 0.620]	< 0.001***

Model characteristics: R^2^ (Nagelkerke) = 0.689, Cohen’s κ = 0.55, Model χ^2^ = 594.49

*MMSE* Mini Mental Status Examination, *CDT* Clock Drawing Test Sunderland, *VVT* Vienna Visuo-constructional Test 3.0 Screening, *HC* Healthy controls, *SCD* Subjective cognitive decline, *MCI* Mild cognitive impairment, *AD* Alzheimer’s dementia, *SE* Standard error

***p < 0.001, Significance at the Bonferroni–Holm adjusted α level

### Associations

The age of participants was moderately correlated to low MMSE scores with *r* = −0.38, [−0.44, −0.30], *p* < 0.001. It had a weak to moderate correlation to low scores in the CDT (*r* = −0.28, [−0.36, −0.21], *p* < 0.001) and low scores in the VVT 3.0 Screening (*r* = −0.28, [−0.36, −0.21], *p* < 0.001). The MMSE rendered a correlation of *r* = 0.31, [0.23, 0.38], with *p* < 0.001 to years of education of participants, the CDT was weakly correlated with *r* = 0.18, [0.11, 0.26], and *p* < 0.001 and the VVT 3.0 Screening correlated moderately with years of education (*r* = 0.31, [0.24, 0.38], *p* < 0.001).

### Assessment of disease progress and prediction

In all, 117 participants (53 men, 64 women) participated in a follow-up 12–48 months (M = 24.02, SD = ±10.1) after the original testing. They had had a mean age of 67.5 (±8.6; range 51–85) years at the original examination and averaged 13.08 (±4.53; range 8–23) years of education. For 74.3% of the participants, the diagnostic group was the same for the original examination and the follow-up, 6% had reached an improvement, 19.7% showed disease-progression. The McNemar–Bowker test of the 4 × 4 contingency table was significant with χ^2^ (2) = 14.250 and *p* = 0.001. Patient flow can be seen in Fig. 1.

Test scores in all three tests dropped between the first and the second examination. Participants scored an average of 27.56 (±2.604) Mdn = 28 (IQR = 27–29) points in the MMSE during the first appointment, they only scored 26.49 (±3.973; Mdn = 28, IQR = 25.5–29) points in the follow-up. CDT scores dropped as well: 9.04 (±1.423; Mdn = 10, IQR = 8.5–10) to 8.56 (±2.255; Mdn = 10, IQR = 8–10) as did VVT 3.0 Screening scores which fell from 9.26 (±1.168; Mdn = 10, IQR = 9–10) points to 9.05 (±1.517; Mdn = 10, IQR = 9–10) in the follow-up. Wilcoxon signed-rank test showed that tests results in all three tests were significantly higher in the baseline than in the follow-up (MMSE: T = 865, Z = −4.750, *p* < 0.001, r = −0.43; CDT: T = 641, Z = −2.053, *p* = 0.020, r = −0.19; VVT 3.0 Screening: T = 514, Z = −1.849, *p* = 0.032, r = −0.18).

In a further set of analyses within the group of returning participants, those who had converted to the next prodromal phase of AD or AD (converter *n* = 23) were compared to stable participants (*n* = 94) to see if there were measurable differences between groups that could be used to predict progress of disease in the future. Mann–Whitney U test showed that converters were significantly older (Mdn = 73), than stable participants (Mdn = 68), *U* = 793.5, *z* = −1.974, *r* = −0.18, *p* = 0.024 (one-tailed). They also had fewer years of education (Mdn = 10) than stable participants (Mdn = 13); this effect was however not significant, *U* = 839.5, *z* = −1.669, *r* = −0.15, *p* = 0.048. The original test scores also did not differ significantly between converters and stable participants (MMSE: *U* = 896.5 (z = −1.290), *r* = −0.12, *p* = 0.1; CDT: *U* = 972.5, *z* = −0.816, *r* = −0.08, *p* = 0.210; VVT 3.0 Screening: *U* = 925.0, *z* = −1.203, *r* = −0.11, *p* = 0.116). Distribution of sex was not significantly different between converters and stable participants either, χ^2^ (1) = 0.074, *p* = 0.786, Odds ratio (OR) = 1.135.

While the stable participants from the original SCD group (*n* = 15, 9 converting, 6 stable) scored an average of 28.33 (±1.21) points in the MMSE, 9.33 (±0.82) in the CDT, and 9.67 (±0.52) in the VVT 3.0 Screening, the converting participants scored an average of 29 (±1.23) points in the MMSE, 9.67 (±0.71) in the CDT, and 9.89 (±0.33). A ROC curve was calculated for the original MCI group (*n* = 76). Progression was set as positive condition. The MMSE showed an AUC of 0.808 (proposed cut-off 27.5, sensitivity 78.6%, specificity 71.0%, YI = 0.495, LR+ = 2.72, LR− = 0.30, PPV = 0.38, NPV = 0.94). The CDT had an AUC of 0.497, the VVT 3.0 Screening had an AUC of 0.488 so no further parameters were calculated.

### Exploring bias

To explore possible bias, returning participants were compared to one-time participants. Mann–Whitney U test revealed that returners were significantly younger than one-time participants at the original testing (*U* = 21,806.0, *z* = −4.420, *p* < 0.001,* r* = −0.18) and had significantly more years of education than non-returners (*U* = 33,163.5, *z* = 2.093, *p* = 0.036 (two-tailed), *r* = 0.08). Returners also scored significantly higher in all three of the neuropsychological tests (MMSE: *U* = 41,329.5, *z* = 6.766, *r* = 0.271, *p* < 0.001; CDT: *U* = 40,682.0, *z* = 6.584, *r* = 0.264, *p* < 0.001; VVT 3.0 Screening: *U* = 37,617.5,* z* = 4.829,* r* = 0.194, *p* < 0.001). There was no significant association between the sex of participants and their return for a second testing, as shown by the chi-squared test, χ^2^ (1) = 0.016, *p* = 0.9, odds ratio = 0.974.

## Discussion

The goal of the presented analysis was to compare the ability of a compilation of visuo-constructional tests, the VVT 3.0 Screening, to spot dementia and its prodromal phases and also to predict disease progress to that of two well established tests: the CDT and the MMSE. Our analyses revealed that the VVT 3.0 Screening and the CDT perform equally well in allocation of patients to diagnostic groups, while both are outperformed by the MMSE. None of the three tests was able to reliably predict disease progression, while the MMSE showed some ability in identification of MCI patients that will progress to AD.

### Diagnosis of AD and prodromal phases

The purely visuo-constructional VVT 3.0 Screening reached a moderate sensitivity of 62.1% and a specificity of 83.1% within this analysis for the discrimination of AD from all other participants and thus has a moderate ability to tell the two groups apart. Results for the CDT were comparable (sensitivity 69.4%, specificity 79.9%). Both tests were, however, outperformed by the multidomained MMSE, which reached a high sensitivity of 94.5% and a specificity of 88.8% (cut-off 25.5). Accordingly, it can be said that although visuo-construction has been shown to be a valid diagnostic tool for AD and sometimes MCI, the MMSE as a multidomain approach is a more accurate tool to date. This supports recommendations on dementia screening in current guidelines [2].

When examining the ability of the tests to discriminate MCI from non-MCI (excluding AD patients), it became obvious that none of the tests performs reliably in this task. As this problem has been identified before [8–10, 20], we suppose that the difficulty herein might be due to the very heterogeneous disease pattern of MCI [16]. As MCI is not AD-specific but can also remain stable, regress or be a precursor of any other form of dementia, it may have a different accentuation in impairment pattern. Considering this, it is also no surprise that especially the single-domain tests are an imprecise tool to spot MCI.

Multinominal logistic regression analyses confirmed these results. For each of the three tests, only the diagnostic categories AD and MCI were used within the model, which shows that the groups HC, SCD, and MCI render results too similar within these tests to use them as a discriminatory tool. This seems plausible especially because there is not much variance in cognitive functions within the group of healthy individuals. SCD is defined as a stage without objective cognitive decline and MCI is known to have a heterogeneous pattern [4, 16]. In the current analysis, 60.6% of participants were allocated to the correct group by the VVT 3.0 Screening. The measure for agreement between clinical diagnosis and diagnosis by VVT 3.0 Screening, Cohen’s κ was 0.28, which means a low agreement. Valencia & Lehrner reached similar results in a precursor study, allocating 60.7% of patients to the right diagnostic group with a low Cohen’s κ of 0.25 [13]. Although the MMSE performed better allocating 74.9% of patients to the correct group (medium Cohen’s κ = 0.55) and the CDT correctly allocated 60.7% (Cohen’s κ = 0.30), none of these suffice to recommend any of the three tests for diagnostic purposes.

Considering these results all of the three tests have some ability to detect AD from all other participants, the MMSE outperforming the single-domain visuo-constructional tests, while none of the three tests can be recommended to discriminate all four groups.

One reason for the latter might be ceiling effects. While the maximum score within the VVT 3.0 Screening is 10 points, the average scores of participants in the HC, the SCD, and the MCI group were above 9 points in the current study, while participants in the AD scored an average of 6.90 (±2.76) points. It seems that deterioration measured by these test scores is not linear along the prodromal phases, leading to very high scores in the groups HC, SCD, and MCI and a lack of sensitivity for minor deterioration of visuo-constructional skills. One potential solution to this problem could be different scoring criteria that are more sensitive to small mistakes.

### Use of single-domain tests

As both purely visuo-constructive tests were outperformed by the multimodal MMSE, this analysis leads to the question whether visuo-construction is possibly not sufficient as the only domain to enable a detailed discrimination between AD and all other participants. Maybe the clinical picture of AD is too diverse to map it assessing only one domain, as Valencia & Lehrner (2018) also suspected [13]. If the VVT 3.0 Screening was to be used in a screening context though, they proposed the use of the alternative cut-off 9.5, considering that the sensitivity plays a more important role than specificity in a screening context. In the current analysis, a shift of the cut-off to 9.5 rendered a sensitivity of 84.9% with a specificity of 55.2%. The use for purposes of screening is thus conceivable. In certain situations, there could also be valid reasons to choose the VVT 3.0 Screening over the MMSE: The MMSE takes about 11 min to apply according to Folstein et al. [6] and requires sufficient language skills on both the patients’ and the examiners’ sides to usher and understand the commands. The VVT 3.0 Screening and CDT on the other hand have the advantage of being very quick to administer—they take only a few minutes—which can be a substantial advantage in a clinical setting. Both tests are also easy to understand and independent from speech comprehension and native language, and thus allow use in settings with substantial language barriers.

As the VVT 3.0 Screening has shown valid ability to detect visuo-constructional deterioration, it could also be worthwhile to investigate its application in differentiation of other forms of dementia. Testing of visuo-constructive functions is recommended for suspected dementia caused by Parkinson’s disease and Lewy body dementia for example [2]. Also, guidelines discriminate between four groups of AD: the anamnestic variant and non-anamnestic variants of a speech-, executive- or visual-constructional-focused kind. A visuo-constructional test like the VVT 3.0 Screening could be helpful to discriminate between these subtypes of AD.

One should also keep in mind that it is no surprise that neuropsychological tests fail to detect SCD, as it is defined as a state that is subjectively perceived as deterioration but cannot be objectified by neuropsychological tests.

### Age as a confounding variable

Furthermore, age correlated moderately with low VVT 3.0 Screening scores and with CDT and MMSE scores. This is not surprising, as age is a risk factor for dementia [29]. All tests also showed a significant correlation to the participants’ education which also aligns with current knowledge, as education was shown to be a protecting factor [30]. Considering this, it could be debated to either add scores for age and education to the VVT 3.0 Screening—a method used within the MoCa for example—or use age- and education-corrected and standardized normal values for interpretation of results.

### Prediction of disease progression

Assessment of the three tests’ ability to predict disease progression was the second goal of this analysis. Data shows that participants reached significantly lower results in all three neuropsychological tests in the second measurement (Table 4). As the pretest likelihood of cognitive symptoms was rather high, since participants presented for neuropsychological testing and some age-related cognitive decline is normal, this result is not surprising. Furthermore, 19.7% of returning patients showed a conversion of their disease within the prodromal phases from the first to the second appointment. This deterioration was significant and indicates that the disease progressed along prodromal phases over time and aligns with the guidelines defining dementia as a progressive decline in cognitive function [2, 5].

**Table 4:** Neuropsychological test scores between tests in the group of returning participants (*n* = 117)

	Baseline	Follow-up
MMSE** Mdn (IQR)	27.56 ± 2.60 28 (27–29)	26.49 ± 3.97 28 (25.5–29)
CDT* Mdn (IQR)	9.04 ± 1.42 10 (8.5–10)	8.56 ± 2.26 10 (8–10)
VVT 3.0 Screening* Mdn (IQR)	9.26 ± 1.17 10 (9–10)	9.05 ± 1.52 10 (9–10)

*MMSE* Mini Mental Status Examination, *CDT* Sunderland Clock Drawing Test, *VVT 3.0 Screening* Vienna Visuo-constructional Test 3.0 Screening, *Mdn* Median, *IQR* Interquartile ratio, Test scores are reported as Mean ± standard deviation and Mdn (IQR), scores compared using Wilcoxon signed-rank test

*p < 0.05, **p < 0.01

An important goal within dementia research is the identification of MCI patients at-risk for development of AD to provide intensified care and enable research on risk factors and causes of conversion. Within the MCI group (*n* = 76), some (*n* = 7) participants improved and were allocated into the SCD group after the second measurement, while 14 participants were diagnosed with AD at the second timepoint. As the mean interval between measurements in the current study was 24.02 (±10.1) months, the annual conversion rate from MCI of AD was about 9%. This aligns with conversion rates found by Langa & Levine in a review [31]. Neither participants from the HC nor from the SCD group conversed to AD between the measurements, which supports the notion of MCI as a symptomatic prodromal state of AD [3, 5]. When comparing participants who did or did not progress between measurements, converters were significantly older than stable participants but did not differ significantly in education, sex, or test scores. The MMSE had some ability to predict conversion within the group of MCI. For a cut-off of 27.5 points (sensitivity 78.6%, specificity 71.0%), test results might be helpful to get a hint of future progress. Nonetheless, none of the three tests nor any other explored variables are sufficient to reliably predict disease progression.

Valencia & Lehrner already proposed that the observation period in this analysis might be too short to observe a sufficient number of patients converting from one stage into another [13] because it has been suggested that AD develops over a period of many years or even decades before it is clinically observable. Another problem when trying to predict disease progression might be that multiple factors like education, occupational situation, and premorbid intelligence may influence test values. Some scientists have also proposed the notion of a cognitive reserve that allows patients to maintain normal cognitive function, while exhibiting disease-related brain pathology up until a ‘tipping point’ is reached [30]. In addition, normal age-related decline might be ‘fogging’ the picture. As all participants decline with age, it is not enough to find decline to predict dementia, but one needs to discriminate normal to ‘more than normal’ decline in order to diagnose a patient. So maybe, the rate of cognitive decline should be assessed instead of comparing absolute test scores.

#### Drop-out bias

When exploring possible drop-out bias, it was shown that returning participants were significantly younger and had received more education than non-returners. They had also scored higher in the MMSE, the CDT, and the VVT 3.0 Screening at the original testing. These numbers point to a certain drop-out bias that may have led younger and ‘fit’ participants to return for a second measurement, while older and more severely impaired patients might have experienced certain hindrances that prohibited them from coming back. In the future, it could be worthwhile to gather information about drop-out reasons.

## Limitations

While the sample recruited for the cross-sectional analysis was large, subgroups were unevenly balanced. This was caused by inclusion bias, as participants had been referred to or visited the neuropsychological department for testing. Consequently, the AD and the MCI groups were significantly larger than the proportion of healthy participants. Furthermore, experimental groups differed in the variables age and years of education. As age has been shown to be a risk factor for the development of AD while education is a protective factor [2], these variables should be controlled for in further studies, e.g., by a matching process. Alternatively, an analysis of covariance (ANCOVA) could be used to computationally eliminate bias. This requires either a large sample (in all experimental groups) or normally distributed data and homogeneity of variances.

## Conclusion

The single-domain Vienna Visuo-constructional Test Screening 3.0 Screening (VVT 3.0 Screening) and Sunderland clock Drawing Test (CDT) showed equal and moderate ability to discriminate between Alzheimer’s Disease (AD) and other participants, but they were outperformed by the Mini Mental State Examination (MMSE). In certain situations, e.g., when time is sparse or language is an issue, the VVT 3.0 Screening can be a short and easy alternative nonetheless. Also the VVT 3.0. Screening could possibly be improved solving the limitations to this study: ceiling effects and confounding influence of age and education. None of the tests could adequately predict membership to all four groups – Healthy controls (HC), subjective cognitive decline (SCD), mild cognitive impairment (MCI), and AD. None of the tests can be recommended to reliably predict disease-progress, solely the MMSE showed some validity in this task.

## Footnotes

1 For detailed information on the VVT 3.0 Screening refer to: psimistri. Vienna Visuo-konstruktiver Test (VVT 3.0)—ein Verfahren zur Bestimmung der Visuokonstruktion. Manual, www.psimistri.com; 2022. The VVT 3.0 and VVT 3.0 Screening can be accessed via www.psimistri.com.
